# Exploring the association between low birth weight and different forms of child malnutrition: A multi-country propensity score matching analysis in South Asia

**DOI:** 10.1371/journal.pone.0358877

**Published:** 2026-09-21

**Authors:** Md Parvej Hussain, Mohammod Mahmudur Rahman, Md Rezaul Kader, Md Robiul Islam Talukder, Md Tareq Ferdous Khan, Foyez Ahmmed

**Affiliations:** 1 Department of Economics, Chittagong University, Chittagong, Bangladesh; 2 Department of Statistics, University of Dhaka, Dhaka, Bangladesh; 3 Department of Biostatistics and Data Science, University of Kansas Medical Center, Kansas City, Kansas, United States of America; 4 Department of Public Health Sciences, College of Behavioral, Social and Health Sciences, Clemson University, Clemson, South Carolina, United States of America; 5 Clemson University School of Health Research, Clemson, South Carolina, United States of America; 6 Department of Statistics, Comilla University, Kotbari, Cumilla, Bangladesh; Jahangirnagar University, BANGLADESH

## Abstract

This study investigated the association between low birth weight (LBW) and various forms of child malnutrition across five South Asian countries (Bangladesh, India, Pakistan, Nepal, and the Maldives). Data from the most recent demographic and health surveys were analyzed. Propensity score matching was applied to evaluate the association between LBW and five forms of child malnutrition: stunting, wasting, underweight, at least one form of child malnutrition (AOFOCM), and multiple concurrent forms of child malnutrition (MCFOCM). Subgroup analyses were performed to assess variability in effects across the countries studied. Analysis of 147,332 complete cases revealed an overall LBW prevalence of 16.23%, with country-specific prevalences of 13.67% (Bangladesh), 16.32% (India), 18.97% (Pakistan), 11.94% (Nepal), and 12.86% (Maldives). Regression analyses of matched samples revealed significant associations between LBW and all five forms of malnutrition. Subgroup analyses also revealed a significant association between LBW and all five forms of malnutrition across the five countries, with the exception of wasting, for which no statistically significant association was observed in Bangladesh and the Maldives. These findings emphasize the importance of context-specific interventions targeting LBW to reduce child malnutrition in South Asia, while taking the observational design and cross-country variability into consideration.

## Introduction

A healthy child is indispensable for building a future generation that is productive, efficient, and able to positively contribute to society and global development [1,2]. However, the different forms of malnutrition, including stunting (too short for age), wasting (too thin for height), overweight, and underweight, pose a significant threat to child health and may even lead to mortality [3–5]. In 2024, child malnutrition increased for the sixth year in a row in the world’s most vulnerable regions [6]. According to the 2025 edition of the UNICEF/WHO/World Bank Group Joint Malnutrition Estimate, in 2024, 150.2 million children under five years of age were affected due to stunting, 42.8 million suffered from wasting, and 35.5 million were overweight [7]. These figures revealed that the 2025 World Health Assembly (WHA) global nutrition targets and the 2030 Sustainable Development Goal (SDG) 2 targets were not on track to be met worldwide [7]. The scenario is particularly concerning in South Asia [8–10], which has the highest wasting prevalence of any sub-regions globally and accounts for half of all children affected by this condition [8]. South Asia also has one of the highest proportions of children affected by stunting, alongside some African regions [11]. It is therefore very crucial for South Asia to address the factors associated with malnutrition in the region. Prior research has identified numerous socioeconomic factors and child health-related issues that may pose a risk for malnutrition in children under the age of five. Among them, some notable factors include maternal nutritional status, maternal education, wealth index, mother’s age at birth, paternal occupation, age of the child, antenatal care, short birth spacing, birth order in the family, etc. [12–20]. Apart from these, low birth weight is indicated as an important determinant of malnutrition in numerous studies [21–25]. South Asia has a higher percentage of low birth weight compared to the other regions, accounting for close to half of all low birthweight newborns in 2020 [26]. Therefore, assessing the association between low birth weight and malnutrition in South Asian countries has been a key interest for researchers aiming to improve child health and development [21,23,27,28].

Low birth weight is considered a useful indicator not only for child malnutrition but also for poverty, maternal health, and healthcare delivery [29]. According to the World Health Organization (WHO), low birth weight is officially defined as birth weight of less than 2500 grams or approximately 5.5 pounds [30]. Compared to children with normal birth weight, those born with low birth weight experience increased rates of subnormal growth, health issues, and poorer neurodevelopmental outcomes. Moreover, the likelihood of adverse outcomes rises as birth weight falls [31]. In 2020, around 20 million babies were born globally with low birth weight [32,33], and more than 40% of these births occurred in South Asian countries [26,33]. Low birthweight affects a large percentage of all live births in South Asia, which is one of the highest rates in the world [26,33], making it a particularly concerning issue in the countries of South Asia. More specifically, across India, Pakistan, and Bangladesh, low birth weight is consistently linked to a higher risk of childhood malnutrition, with increased odds of stunting, wasting, and underweight conditions [21,23,27]. It is important to note that there are many factors associated with malnutrition that also may affect the weight of children at birth, especially those related to social and maternal health. For example, mothers’ education, mothers’ age at birth, wealth index, and antenatal care (ANC) are also mentioned as potential predictors of low birth weight in previous studies [34–38]. Considering these issues, in this study, our motivation is to observe the association between low birth weight and malnutrition in children under five years of age.

Previous studies have employed various statistical models and techniques to identify the significant determinants of child malnutrition across different regions. The most common and widely used method for this type of research is the multiple binary logistic regression model, while some studies also used the ordinal logistic regression model [12,24,39–42]. One of the limitations of using these methods in observational studies is that they are often susceptible to confounding bias, which arises when a risk factor for the outcome also affects the treatment of interest [43]. To overcome this problem, propensity score matching (PSM) can be utilized as an alternative method [20,44,45]. The PSM assesses the association of the treatment variable with the outcome [20] by balancing the covariates between treated and untreated groups. Our study addresses that issue. We utilize the propensity score matching approach in malnutrition research by examining the association between LBW and various forms of child malnutrition across five South Asian countries to minimize confounding bias. In addition, this study investigated the association between LBW and five different forms of child malnutrition in the literature utilizing five nationally representative data sets from five South Asian countries. Unlike most existing studies, which are typically limited to a single country or a narrower set of outcomes, our analysis provides a broader and more comprehensive assessment while minimizing confounding bias.

## Methods

### Study design and participants

This study used data from the most recent Demographic and Health Survey (DHS) conducted in five South Asian countries, including Bangladesh, Nepal, India, Pakistan, and Maldives. The DHS are nationwide household surveys that use standardized questionnaires to collect data on a wide range of health and nutrition indicators, with a primary focus on mother and child health [46]. The DHS program employed an identical survey design across all countries, consisting of a cross-sectional approach and a multistage stratified cluster random sampling strategy, ensuring that the data were nationally representative of each country’s population [46,47]. For further detailed information regarding the survey methods, we refer to the DHS reports for the countries under the study [48–52].

### Data extraction and preparation

**Fig 1:**
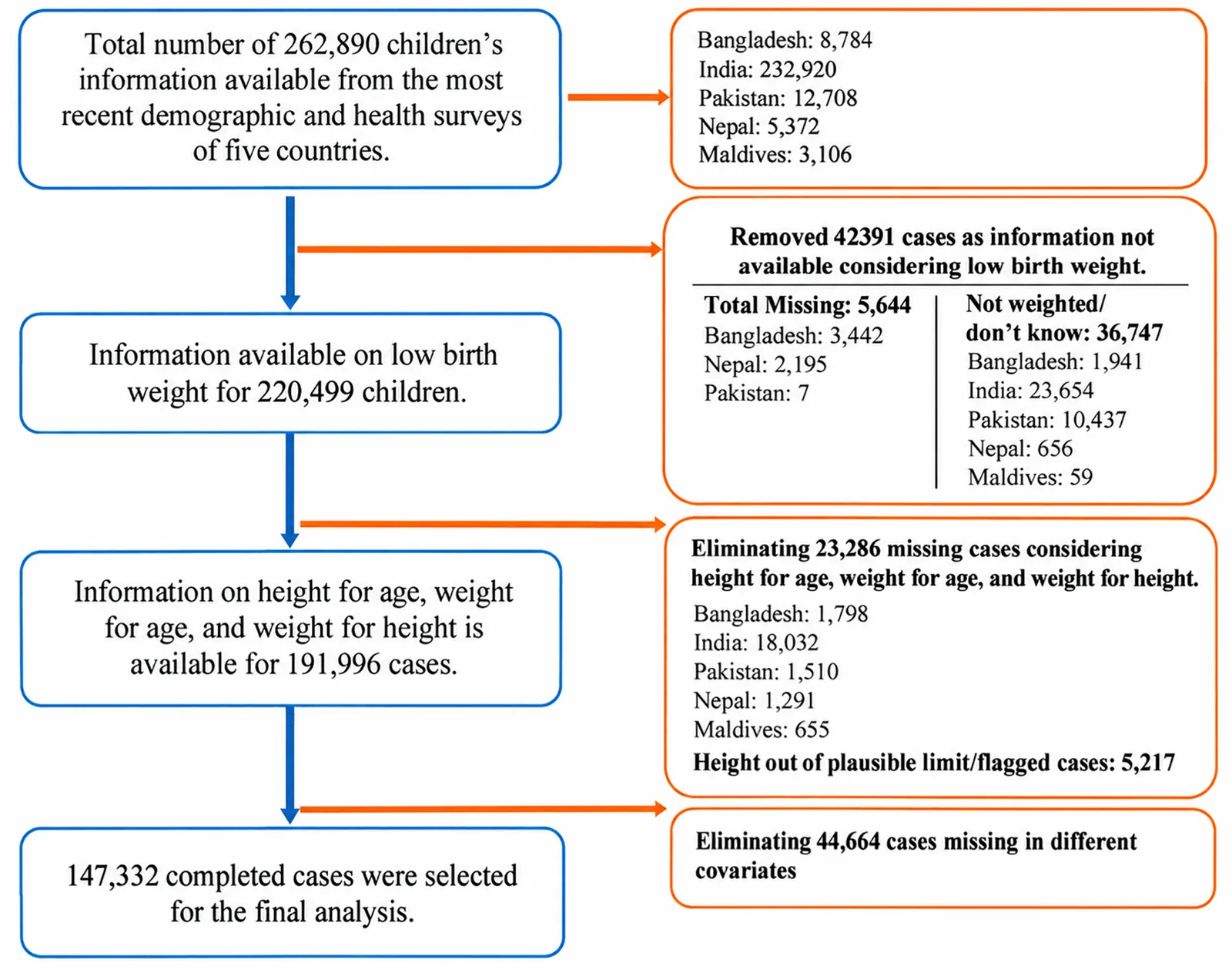
Flow chart explaining how the sample was extracted for final analysis.

The most recent DHS datasets from five South Asian countries, including Bangladesh (BDHS 2022), Nepal (NDHS 2022), India (IDHS 2019-21), Pakistan (PDHS 2017-18), and Maldives (MDHS 2016-17) were extracted from the DHS program’s official database following approval of a data-use request [53]. The initial combined datasets across these countries included health information for 262,890 children. We removed cases that did not contain children’s birth weight data, including those marked as ‘Not weighted’ or ‘Don’t know’, as well as missing entries. After removing these, we were left with 220,499 children’s records with valid low birth weight information. From this subset, we focused on additional health information such as height-for-age, weight-for-age, and weight-for-height, which were available for 191,996 children. After further excluding cases with missing values in various covariates, the final dataset had a total sample of 147,332 children. A detailed overview of the exclusion process is presented in the flowchart in Fig 1.

### Outcome variables

This study assessed nutritional status using three anthropometric measurements: height-for-age, weight-for-height, and weight-for-age, based on World Health Organization (WHO) growth standards [54]. These anthropometric measurements were collected at the time of the survey among children under five years of age (0–59 months). Using these indices, we constructed five binary response variables: stunting, wasting, underweight, AOFOCM (at least one form of child malnutrition), and MCFOCM (multiple concurrent forms of child malnutrition). The height-for-age index measures a child’s height compared to what is expected for their age, according to the standard growth chart [54]. Children whose height-for-age was more than 2 standard deviations (SD) below the median of the reference population were categorized as “stunted” (Yes/No). Similarly, wasting and underweight were determined using weight-for-height and weight-for-age indices, respectively, with binary categorization (Yes/No) based on the −2 SD cutoff [54]. Finally, the outcome variable AOFOCM was defined as ‘Yes’ if a child exhibited at least one positive indicator (stunting, wasting, or underweight), and ‘No’ otherwise [55]. In contrast, MCFOCM was defined as ‘Yes’ if a child exhibited at least two of the aforementioned malnutrition indicators, and ‘No’ otherwise [55].

### Treatment and covariates

Low birth weight (LBW) was used as the treatment variable. Following WHO guidelines, LBW was defined as a binary variable, with children weighing less than 2,500 grams classified as ‘Yes’ (abnormal weight) and those weighing 2,500 grams or more classified as ‘No’ (normal weight) [30]. In the DHS surveys, birth weight is obtained retrospectively, based on either recorded birth weight (from health cards) or maternal recall at the time of the survey. To control potential confounding effects, several other variables were also included in the analyses. These variables include country (Bangladesh, India, Maldives, Nepal, Pakistan), mother’s age (<20, 20–34, >=35), mother’s education (No education, Primary, Secondary, Higher), wealth Index (Poorest, Poor, Middle, Rich, Richest), sex of child (Male, Female), ANC (<4, >=4), intake of iron tablet/syrup (No, Yes), C-section (caesarean section) (No, Yes), place of residence (Urban, Rural), exposed to media (No, Yes), BMI (body mass index) (Underweight, Normal, Overweight, Obese), birth order (1–2, 3–4, Higher), terminated pregnancy (No, Yes) and EIBF (early breast feeding) (No, Yes). The variable “exposure to media” was determined by the mother’s engagement with watching television, reading newspapers or magazines, or listening to the radio. Mothers who reported exposure to at least one of these media sources were classified as exposed to media [20]. As noted by one of the reviewers, it is important to elaborate on the relevance of this covariate. Maternal exposure to media is particularly relevant in this context because previous studies have shown that it is associated with a reduced risk of child malnutrition, including stunting, wasting, and underweight [56–58]. Moreover, exposure to media has been associated with greater maternal healthcare utilization in the south Asian countries after adjusting for several socioeconomic factors [59]. BMI was categorized according to the recommended measures for Asian people: underweight (<18.5 kg/m^2^), normal (18.5 kg/m^2^ to < 23 kg/m^2^), overweight (23 kg/m^2^ to < 27.5 kg/m^2^), and obese (≥ 27.5 kg/m^2^) [60].

### Variable to test heterogeneous effects

Although the primary objective of this study was to assess the association between LBW and various forms of child malnutrition, the magnitude of this association may vary across countries, an aspect known as heterogeneous effects. To assess this, we examined whether the association between LBW and each form of child malnutrition differed across five national subgroups: Bangladesh, Nepal, India, Pakistan, and the Maldives.

### Statistical analysis

To evaluate the association between the treatment variable (LBW) and the outcome measures (various forms of child malnutrition), a comprehensive set of statistical methods was employed. Descriptive statistics, including frequencies and percentages, were used to summarize the sample characteristics. Chi-square tests were initially performed to examine the bivariate association between LBW and the outcome variables in the unmatched dataset. Additionally, chi-square tests were also conducted between LBW and each of the covariates to assess the relationship between them and identify any potential confounding effects of the covariates on the treated variable. Significant associations in these comparisons imply the evidence of confounding, which justifies the need for matching in order to reduce confounding bias and estimate the association of LBW with child malnutrition outcomes. All statistical analyses were performed without incorporating the DHS sampling weights, clustering, or stratification.

To control confounding effects, we applied a one-to-one propensity score matching (PSM) approach and constructed a matched sample of treated (LBW) and control (non-LBW) children. PSM aims to reduce confounding bias by balancing the baseline characteristics between the treated and control groups, so that valid conclusions can be made regarding the impact of a treatment on the outcome measures [61]. To estimate the propensity scores, we utilized logistic regression as the distance function, which is the most commonly used approach [62]. The model produces an estimated propensity score (PS) for each individual, representing the predicted probability of being treated given their observed covariates [63]. Once the PSs are calculated, the matched pairs between treated and control subjects were formed using their PSs. The most popular methods for forming matched pairs based on propensity scores are: nearest neighborhood matching (NNM) and optimal matching (OM) [62,64]. However, Gu and Rosenbaum [63] showed that NNM outperformed OM in terms of balancing covariates, and therefore, we employed the NNM algorithm without replacement to match treated individuals with control individuals based on their PSs. NNM selects patients one by one from the treated group. For each treated subject, it finds an observation in the control group whose PS is closest to that treated subject, and includes the observation in the matched sample [62]. No caliper or common support restriction was applied. Given the multi-country nature of the data, exact matching on country was performed to ensure that comparisons were made within the same country.

Once the matched sample is found, we assessed the quality of matching using three approaches. First, we assessed the standardized mean difference (SMD) for all covariates between the matched treatment and control groups, where SMDs less than 0.1 indicate a good matching [62]. Second, we compared the pseudo $R^{\text{2}}$ values and likelihood ratio (LR) test statistics from logistic regression models fitted to the unmatched and matched datasets. The lower pseudo $R^{\text{2}}$ value and the insignificant goodness of fit test suggest evidence of good covariate balance [65]. Finally, we also examined the distribution of PSs between treated and control groups to visually assess the effectiveness of the matching process. The more closely the two distributions overlap, the more similar characteristics the treated and control groups have, which is indicative of more effective matching [20].

After ensuring the data were successfully matched, we estimated the average treatment effect on the treated (ATT) for each outcome variable using three modeling approaches: Linear Probability Model (LPM), Logistic Regression, and Weighted Least Squares (WLS) [61,66,67]. Each model was applied to the matched dataset while adjusting for all covariates to obtain robust estimates of the treatment effect. The post-matching regression models were used as an additional adjustment step to account for any small residual imbalance after matching and to improve the precision of the estimated association within the matched sample. In addition, we conducted unadjusted analyses within the matched sample and checked if the results differ from the adjusted analysis. We also considered the possibility of heterogeneous effects for different countries. Therefore, we performed subgroup analyses by country and estimated association between LBW and all forms of child malnutrition for different countries. Data management and curation were performed using Stata, while the propensity score matching was implemented using the R package “MatchIt” [68].

## Results

### Background characteristics of the respondents

**Fig 2:**
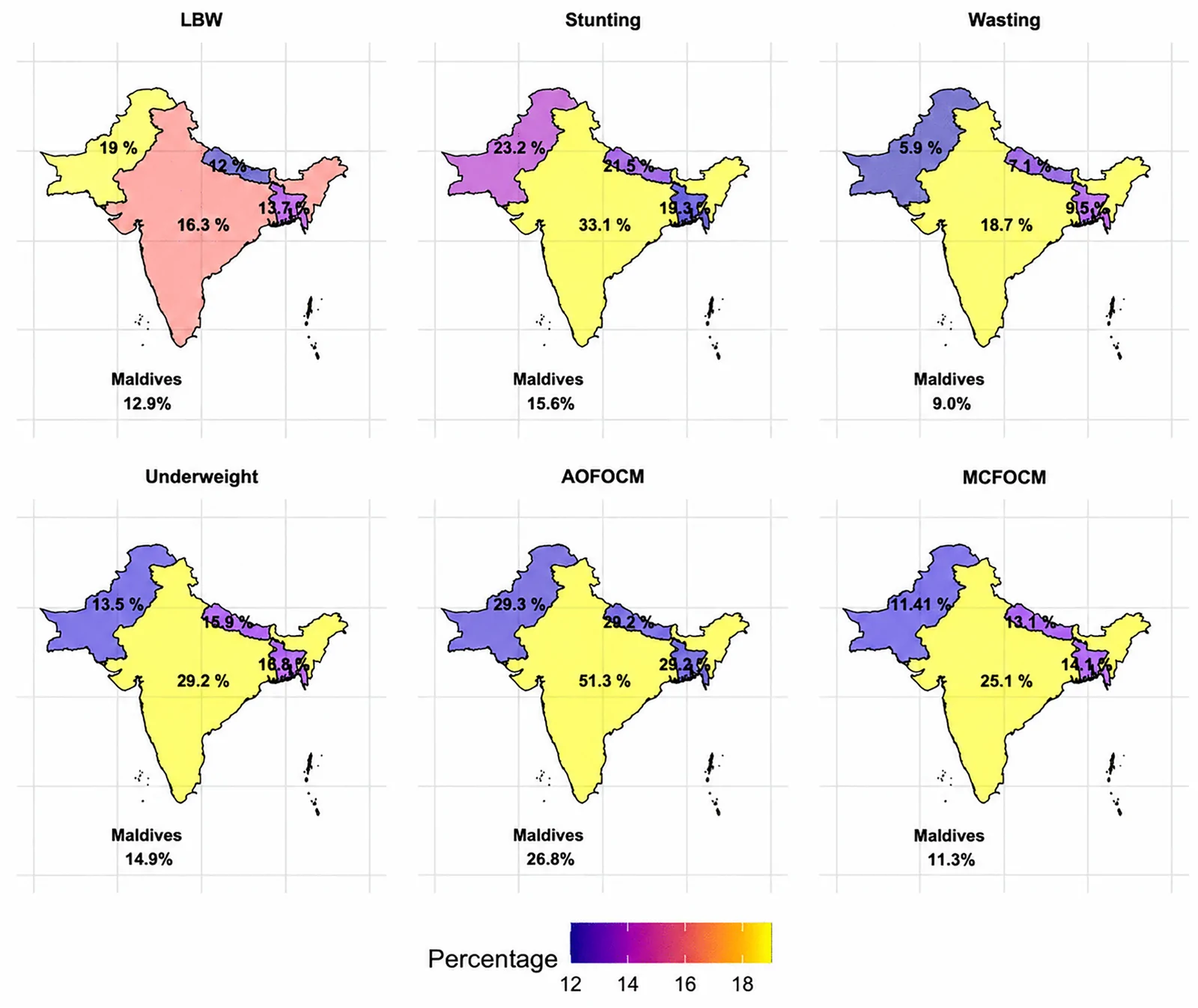
Spatial distribution of low birth weight (LBW), stunting, wasting, underweight, at least one for child malnutrition (AOFOCM), and multiple concurrent forms of child malnutrition (MCFOCM) for the five South Asian countries.

Frequency and percentage distributions of the sociodemographic or potential confounding variables, treatment, and outcome variables are reported in the supplementary Table S1 in S1 File. Among the 147,332 participants, the majority were from India (96.69%), with smaller proportions from Bangladesh (1.04%), Maldives (1.12%), Nepal (0.78%), and Pakistan (0.37%). Most mothers were aged 20–34 years (86.82%), had secondary education (54.10%), and resided in rural areas (77.70%). About 38.15% had fewer than four antenatal care (ANC) visits, while 89.40% reported iron intake during pregnancy, and 23.43% had cesarean deliveries. Regarding household wealth, over 45% belonged to the poorest or poor quintiles. Nearly half of the children were female (46.50%), and early initiation of breastfeeding (EIBF) was reported in 43.90% of cases. Exposure to media (75.99%) and maternal nutritional status varied, with 17.62% underweight and 9.98% obese. The majority of children had a birth order of either 1 or 2 (71.19%), and 15.24% of mothers reported experiencing a terminated pregnancy. The prevalences of low birth weight, stunting, wasting, and underweight were 16.23%, 32.56%, 18.36%, and 28.69%, respectively. Notably, 50.51% of children experienced any form of malnutrition (AOFOCM), and 24.66% had multiple concurrent forms (MCFOCM). The country-wise spatial distributions of the prevalence of LBW and all outcome measures are also presented in Fig 2.

### Bivariate analyses in the unmatched data

In the unmatched sample, significant associations were found between the treatment (LBW) and all forms of outcome variables (stunting, wasting, underweight, AOFOCM, and MCFOCM) (p < 0.001; Table 1). Additional bivariate analyses were conducted to identify potential confounders significantly associated with the treatment variable. All covariates, including country, mother’s age, mother’s education, wealth index, sex of child, ANC visit, taking iron tablet, place of residence, exposure to media, BMI, birth order, terminated pregnancy, and EIBF, were significantly associated with LBW, except for C-section delivery (Supplementary Table S2 in S1 File).

**Table 1:** Association between outcomes and the treatment variable in the unmatched data.

Variables	Level	Low Birth Weight		p-value (Chi-square)
		Yes 23909 (16.23)	No 123423 (83.77)	
**Stunting**	No	14127 (14.22)	85234 (85.78)	<0.001
	Yes	9782 (20.39)	38189 (79.61)	
**Wasting**	No	18522 (15.4)	101759 (84.6)	<0.001
	Yes	5387 (19.91)	21664 (80.09)	
**Underweight**	No	14285 (13.6)	90780 (86.4)	<0.001
	Yes	9624 (22.77)	32643 (77.23)	
**AOFOCM**	No	9333 (12.8)	63577 (87.2)	<0.001
	Yes	14576 (19.59)	59846 (80.41)	
**MCFOCM**	No	15463 (13.93)	95543 (86.07)	<0.001
	Yes	8446 (23.25)	27880 (76.75)	

### Covariate balance using propensity score matching

**Fig 3:**
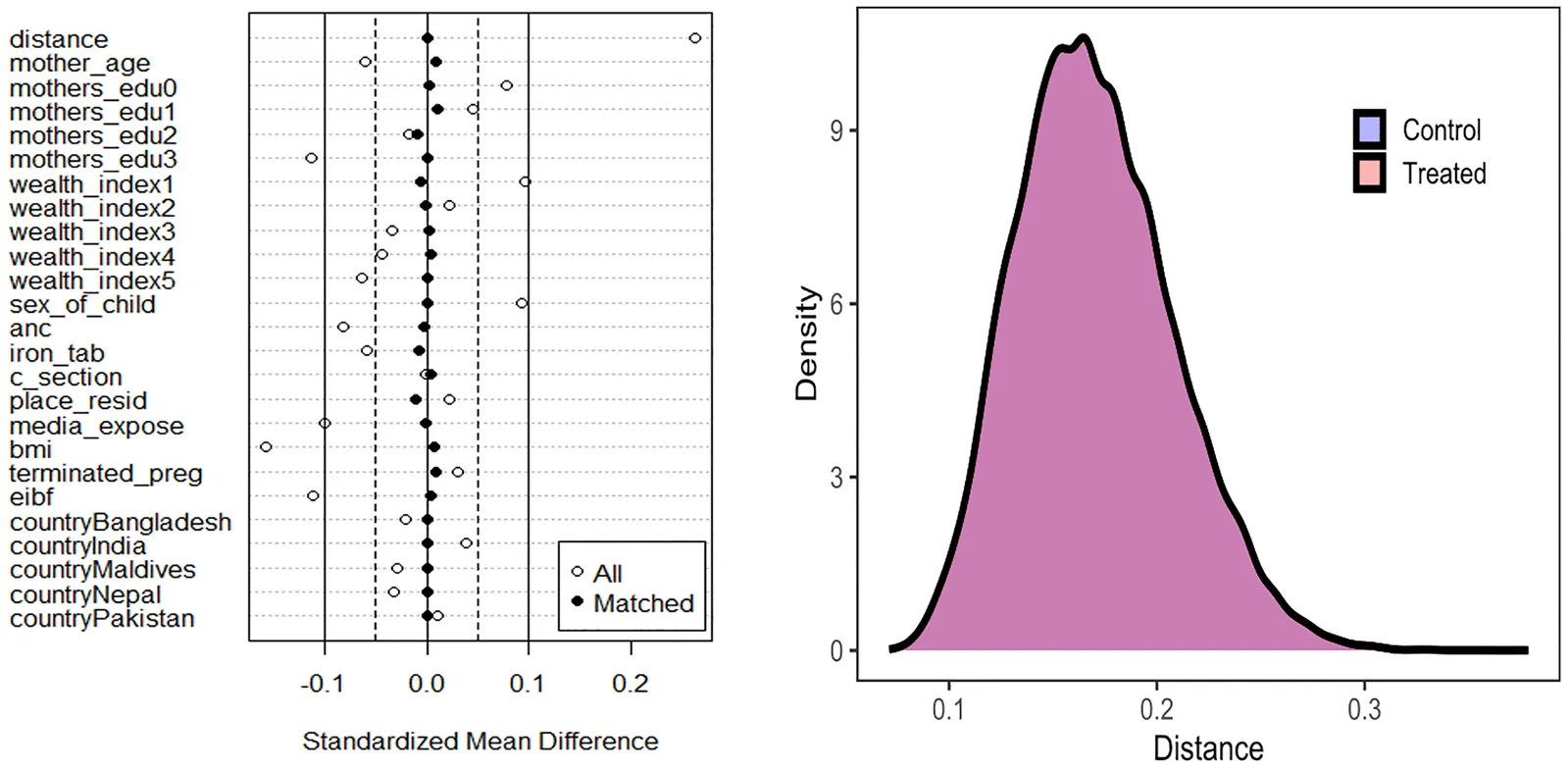
Standardized mean differences (SMDs) for unmatched and matched sample (left), and the distribution of propensity scores for the treated and control groups obtained from the logistic regression (right).

After conducting one-to-one PSM using the distance function based on logistic regression, a matched sample of 47,818 (Bangladesh: 418, India: 46494, Pakistan: 206, Nepal: 276, and Maldives: 424) respondents was obtained. The SMDs between treatment and control groups for all the covariates in the matched sample were much less than 0.1 (Fig 3 and Supplementary Table S3 in S1 File), which implies a good matching and balance of the covariates over the exposed (treated) and unexposed (control) groups. To further assess the quality of the matching, pseudo $R^{\text{2}}$ values and likelihood ratio (LR) test statistics with p-values were computed for logistic regression models fitted separately to unmatched and matched datasets (Supplementary Table S4 in S1 File). We observed that the pseudo $R^{\text{2}}$ value decreased from 0.0112 (pre-match) to 0.0001 (post-match). The LR test indicated that the model with the matched sample had a good fit since it provided a non-significant p-value (p = 0.9975), whereas the model using the pre-matched sample showed poor fit (p < 0.00001). These results further confirm that the matching was good and effectively reduced selection bias. Finally, the distribution of propensity scores for the treated and control groups in Fig 3 shows substantial overlaps, indicating that the children with and without LBW had comparable characteristics.

### Average treatment effects on the treated for various forms of child malnutrition

Table 2 presents the estimated average treatment effects on the treated (ATT) for various forms of child malnutrition, obtained using three statistical models: the linear probability model (LPM), logit model, and weighted least squares (WLS) model. We found the same conclusions (very little difference in the numerical results) in both adjusted and unadjusted analysis, therefore, reported more precise adjusted analysis results for simplicity. All models consistently indicated that LBW significantly increased the risk of child malnutrition. The LPM suggested that children with LBW had significantly (p < 0.0001) higher probabilities of stunting (8.67 percentage points), wasting (4.24 percentage points), underweight (12.52 percentage points), AOFOCM (10.97 percentage points), and MCFOCM (11.38 percentage points) compared to their counterparts. The logistic regression model further confirmed these findings, indicating that children with LBW had significantly higher odds (all p-values <0.0001) of stunting (adjusted odds ratio (AOR) = 1.472), wasting (AOR = 1.304), underweight (AOR = 1.800), AOFOCM (AOR = 1.595), and MCFOCM (AOR = 1.776). The WLS model with covariates produced similar estimated treatment effects as the LPM, reinforcing the conclusion that LBW significantly increased the probability of all forms of child malnutrition. We included both LPM and WLS models to ensure inferential rigor. The LPM is presented for its direct interpretability of marginal effects on the probability scale. However, because binary outcomes inherently violate the ordinary least square assumption of homoscedasticity, we utilized WLS model to obtain efficient estimators and mathematically valid standard errors.

**Table 2:** Average treatment effects on the treated (ATT) for various forms of child malnutrition using linear probability model (LPM), logit model, and weighted least square (WLS) model.

Outcomes		LPM	Logit	WLS
**Stunting**	Treatment	0.0867	0.3867	0.0868
	P-value	<0.0001	<0.0001	<0.0001
**Wasting**	Treatment	0.0424	0.2655	0.0392
	P-value	<0.0001	<0.0001	<0.0001
**Underweight**	Treatment	0.1252	0.5876	0.1216
	P-value	<0.0001	<0.0001	<0.0001
**AOFOCM**	Treatment	0.1097	0.4669	0.1091
	P-value	<0.0001	<0.0001	<0.0001
**MCFOCM**	Treatment	0.1138	0.5742	0.1078
	P-value	<0.0001	<0.0001	<0.0001

### Effect modification by country

**Fig 4:**
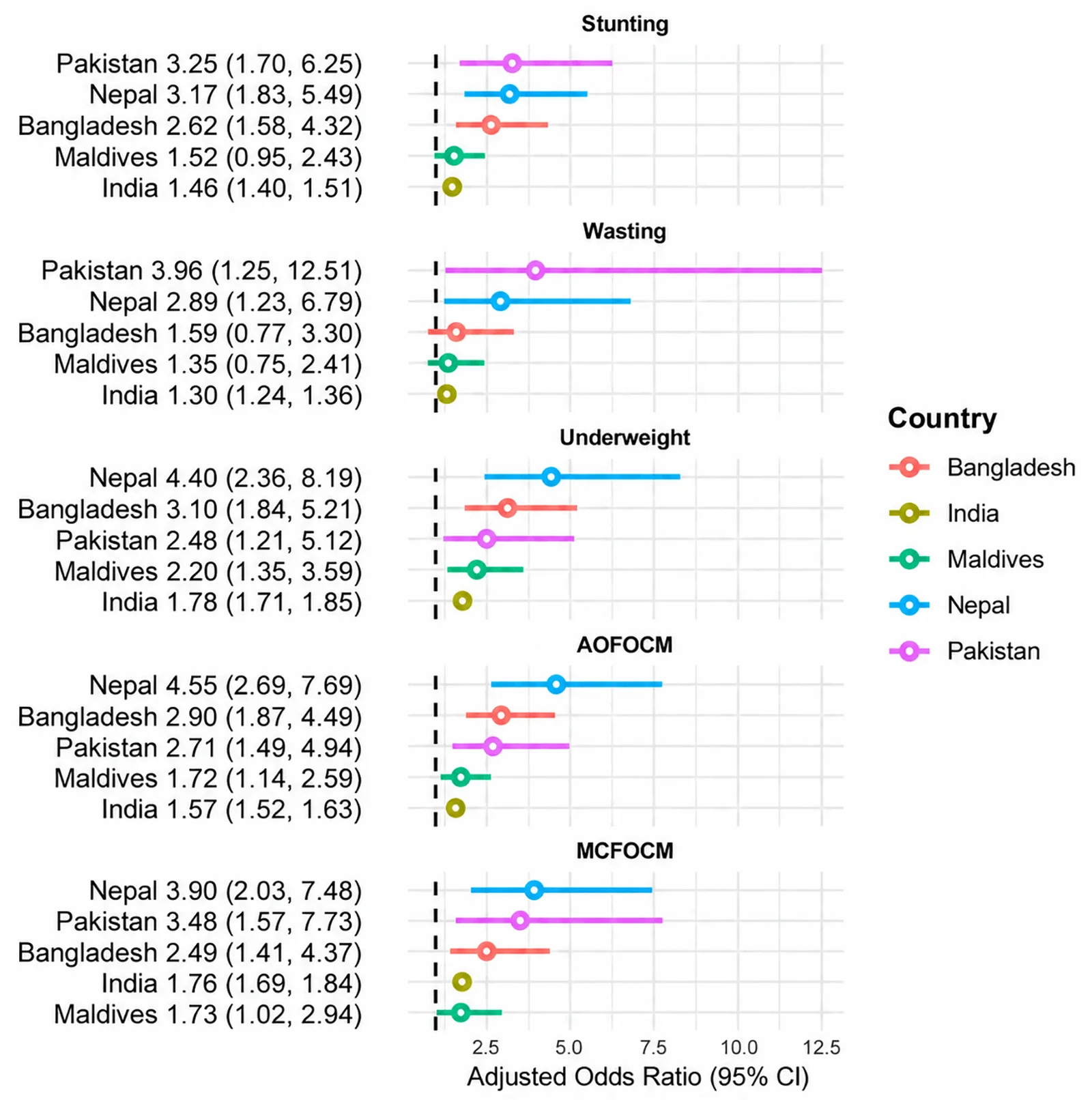
Adjusted odds of different forms of child malnutrition (stunting, wasting, underweight, AOFOCM: at least one for child malnutrition, MCFOCM: multiple concurrent forms of child malnutrition) with confidence intervals for treated (low birth weight) vs control (not low birth weight) by five South Asian countries.

Country-wise subgroup analyses are presented in Supplementary Table S5 in S1 File and visually summarized in Fig 4. Overall, the association between LBW and child malnutrition outcomes showed substantial variation across the five South Asian countries; however, these findings should be interpreted with caution, as India contributed a substantially larger sample compared to the relatively smaller matched samples from the other countries. For stunting, Pakistan showed the highest odds (AOR = 3.254, 95% CI = 1.69–6.24), followed by Nepal (AOR = 3.17, 95% CI = 1.83–5.49), Bangladesh (AOR = 2.615, 95% CI = 1.58–4.32) and India (AOR = 1.46, 95% CI = 1.40–1.51), while the association in the Maldives was not statistically significant. The adjusted odds of wasting were significantly higher among LBW children in Pakistan (AOR = 3.96, 95% CI = 1.25–12.51), Nepal (AOR = 2.89, 95% CI = 1.23–6.79), and India (AOR = 1.30, 95% CI = 1.24–1.36), whereas the association was not significant for Maldives and Bangladesh. A significant association of LBW with underweight was observed across all countries, with the largest effect in Nepal (AOR = 4.40, 95% CI = 2.36–8.19). Elevated odds were also seen in Bangladesh, Pakistan, and the Maldives, while India exhibited the smallest effect size. A similar pattern was observed for AOFOCM, with Nepal reporting the highest odds (AOR = 4.55, 95% CI = 2.69–7.69) and India (AOR = 1.57, 95% CI = 1.52–1.63) had the lowest. Finally, MCFOCM also demonstrated substantial variation across all countries, with the strongest association found in Nepal (AOR = 3.90, 95% CI = 2.03–7.48), followed by Pakistan, Bangladesh, India, and the Maldives.

## Discussion

This study assessed the association between LBW and five distinct forms of child malnutrition across five South Asian countries, using a matched sample obtained through a one-to-one propensity score matching (PSM) approach. The high prevalence of LBW (16.23%), stunting (32.56%), wasting (18.36%), underweight (28.69%), at least one form of malnutrition (AOFOCM, 50.51%), and multiple concurrent forms of malnutrition (MCFOCM, 24.66%) highlights the severity of the public health challenge in the region. Our findings provided robust evidence that LBW is a significant risk factor for child malnutrition in South Asia. Three modeling approaches, including LPM, logistic regression, and WLS with covariate adjustment, consistently demonstrated that LBW significantly increased the risks of stunting, wasting, underweight, as well as AOFOCM and MCFOCM. Country-specific subgroup analyses revealed notable heterogeneity in these associations. Nepal exhibited the highest adjusted odds in underweight, AOFOCM and MCFOCM, while Pakistan demonstrated highest odds in stunting and wasting.

Our findings are consistent with previous large-scale survey-based evidence, particularly from low- and middle-income countries. A pooled study across six South Asian countries showed a significant association between LBW and three forms of child malnutrition outcomes, including wasting, severe wasting, and co-occurrence of wasting and stunting [28]. Similarly, a study covering 32 sub-Saharan African countries reported that children with LBW were significantly more likely to be stunted, underweight, and wasted [24]. Another large-scale study involving 26 sub-Saharan African countries found LBW as a significant determinant of both standalone and coexisting forms of undernutrition [25]. This study considered seven forms of undernutrition outcomes: the three individual indicators (stunting, wasting, underweight), all possible pairwise combinations, and the co-occurrence of all three. Extending this framework, our study also provides new evidence of the association between LBW and two broader indicators (AOFOCM and MCFOCM), which may be relevant in the South Asian context due to their high prevalence. These findings may help prioritize interventions targeting the most vulnerable child cohorts.

Despite substantial heterogeneity in the strength of associations, country-specific subgroup analyses showed that LBW was significantly associated with underweight and the combined malnutrition outcomes in all five countries, whereas its association with stunting and wasting were not statistically significant in some countries. These findings are consistent with previous studies; however, previous studies generally focused on only three malnutrition outcomes, whereas our study assessed five. A large-scale survey-based study in India reported similar associations between LBW and stunting, wasting, and underweight [21]. Several studies in Bangladesh also support our findings [23,55,69,70]. Notably, our study showed that Nepal exhibited the strongest associations considering underweight, AOFOCM, and MCFOCM. While relatively few studies in Nepal have investigated child malnutrition, some evidence links LBW with poor nutritional outcomes and early childhood development indicators [69,71]. In Pakistan, LBW was associated with an increased likelihood of child malnutrition. Although nationally representative evidence remains limited, a provincial study conducted in Sindh reported significant associations of LBW with moderate and severe wasting and with concurrent stunting and wasting [27]. Another study found a high adjusted odds ratio (AOR = 23.34) for severe undernutrition associated with LBW in Pakistan, with statistical significance [69]. The observed cross-country heterogeneity may be explained by differences in healthcare infrastructure, maternal and child nutrition programs, socioeconomic conditions, and childcare practices.

The consistency of our findings across multiple statistical models, combined with rigorous balancing through the PSM approach, reinforces the robust association between LBW and child malnutrition outcomes. Several plausible biological mechanisms may explain why LBW predisposes children to undernutrition. LBW is often a consequence of intrauterine growth restriction or premature birth, both of which interrupt normal fetal development and lead to altered metabolic programming and impaired organ maturation [72–75]. These biological disruptions can compromise nutrient absorption, impair growth hormone regulation, and weaken immune function, increasing susceptibility to infections and limiting postnatal catch-up growth [72–76]. Consequently, children with LBW are at significantly higher risk of stunting, wasting, being underweight, or combinations of these forms of malnutrition [22,23,70]. As noted by the reviewer, child malnutrition is multifactorial and may follow a “causal pathway” involving interconnected determinants. In our study, maternal education, rural residence, and household wealth were associated with LBW, suggesting that socioeconomic disadvantage may increase the risk of LBW, which in turn contributes to child malnutrition. Thus, LBW may act as both an intermediate factor and a marker of broader disadvantage. While our analysis focuses on associations, such causal pathways have to be explored in future studies. However, although it is very plausible that the association between LBW and malnutrition is caused by low socioeconomic status, previous studies from high-income countries including England, South Korea, and Lithuania still report significant association between LBW and adverse health effects in children [77–79].

From a policy perspective, our findings highlight the urgent need for targeted interventions to reduce LBW and mitigate its effects. Increasing antenatal care coverage, improving maternal nutrition (especially iron, folate, and protein intake), and promoting institutional deliveries are evidence-based strategies to reduce LBW prevalence and improve child growth outcomes [80,81]. Furthermore, interventions such as Kangaroo Mother Care and early initiation of breastfeeding have been shown to improve survival and growth among LBW infants [82,83]. Integrating these maternal and child health programs into national nutrition policies could play a vital role in achieving Sustainable Development Goal 2 (SDG-2) and meeting the World Health Assembly (WHA) global nutrition targets.

### Strengths and limitations

This study has several strengths. First, it used the most recent nationally representative DHS survey datasets for the five South Asian countries. Second, five distinct forms of malnutrition outcomes were defined to assess their association with LBW, whereas most previous studies focused on individual outcomes. Third, propensity score matching was used to balance observed covariates between children with and without LBW, thereby reducing selection bias and facilitating comparisons between groups with similar observed characteristics within the observational data. Fourth, multiple modeling approaches were employed to confirm the robustness of the findings. Fifth, country-wise sub-analyses provided comparable results across countries and reinforced the generalizability of the conclusions. Despite several strengths, this study has some limitations. First, although PSM adjusts observed covariates, it cannot account for unmeasured confounders, such as genetic predispositions or intrauterine infections, prenatal nutrition, maternal stress, or environmental exposures. Second, birth weight data in DHS surveys are often based on maternal recall, which may introduce reporting bias [83]. Third, DHS datasets lack detailed clinical information and data on underlying health conditions, which may be important for understanding the mechanisms linking LBW and malnutrition and may require further adjustment. Fourth, although the study used the nationally representative datasets, the findings may not be generalized to other regions and to some underrepresented subpopulations within the studied countries. Fifth, country-specific heterogeneity may result from other factors such as differences in the health system, socio-cultural practices, etc. Failing to control these contextual factors could lead to biased estimates. One of the referees mentions about the consideration of DHS survey design and weights in the analysis. However, in the context of this study, incorporating survey weights is not straightforward. Our analysis combines DHS data from multiple countries, where survey weights are normalized within each country and are not directly comparable across countries. Proper use of these weights in a pooled analysis would require additional methodological considerations and more advanced statistical approaches. While there are established methods for incorporating survey weights in propensity score analyses within a single survey, there is currently no widely accepted approach for integrating complex survey weights into propensity score matching in multi-country pooled settings. In addition, the use of survey weights is more relevant when the goal is to obtain country-level or population-representative estimates, which is not the primary objective of our study. However, we note this as a limitation of this study. Further investigation can be carried out using an appropriate methodology to incorporate survey weight in the current setting. Although we used the most recent DHS survey available for each country, some datasets are relatively old, and the findings may not fully reflect current population. We note that key variables such as birth weight and anthropometric measures in DHS data are often systematically missing (e.g., due to home births or socioeconomic disadvantage). As a result, the analytic sample may overrepresent children from relatively better-off households or those with improved access to healthcare services. This selective inclusion could potentially bias the estimated associations, for example, by underestimating or overestimating the strength of the relationship between LBW and later anthropometric outcomes, depending on the direction of the underlying missingness mechanism.

## Conclusions

In conclusion, our study revealed a strong association between LBW and multiple forms of child malnutrition across South Asia. The findings highlight the urgency of integrated maternal-child nutrition policies that focus on preventing LBW and supporting affected infants, with cross-country approaches tailored to national contexts.

## Supporting information

S1 File Contains Tables S1–S5. (DOCX)
