# Supplementary material for: Exploring the association between low birth weight and different forms of child malnutrition: A multi-country propensity score matching analysis in South Asia
Source: PLoS One. 2026 Sep 21;21(9):e0358877. doi: 10.1371/journal.pone.0358877 (PMC13592607; doi:10.1371/journal.pone.0358877)
Supplement: Supplementary file 1 [file pone.0358877.s001.docx]

Supplementary File of the manuscript “**Exploring the association between low birth weight and different forms of child malnutrition: A multi-country propensity score matching analysis in South Asia**”

Table S1: Frequency and percentage distribution of the selected variables.

| **Variables** | **Level** | **Overall (147332)** |
| --- | --- | --- |
| Country | Bangladesh | 1529 (1.04) |
|  | India | 142455 (96.69) |
|  | Maldives | 1649 (1.12) |
|  | Nepal | 1156 (0.78) |
|  | Pakistan | 543 (0.37) |
| Mother’s Age | <20 | 4170 (2.83) |
|  | 20-34 | 127915 (86.82) |
|  | >=35 | 15247 (10.35) |
| Mother’s Education | No education | 26095 (17.71) |
|  | Primary | 17881 (12.14) |
|  | Secondary | 79701 (54.1) |
|  | Higher | 23655 (16.06) |
| Wealth Index | Poorest | 33878 (22.99) |
|  | Poor | 33524 (22.75) |
|  | Middle | 30106 (20.43) |
|  | Rich | 27215 (18.47) |
|  | Richest | 22609 (15.35) |
| Sex of Child | Male | 78818 (53.5) |
|  | Female | 68514 (46.5) |
| ANC | <4 | 56214 (38.15) |
|  | >=4 | 91118 (61.85) |
| Intake Iron Tablet/syrup | No | 15621 (10.6) |
|  | Yes | 131711 (89.4) |
| C-Section | No | 112809 (76.57) |
|  | Yes | 34523 (23.43) |
| Place of Residence | Urban | 32853 (22.3) |
|  | Rural | 114479 (77.7) |
| Exposed to Media | No | 36842 (25.01) |
|  | Yes | 110490 (74.99) |
| BMI | Underweight | 25966 (17.62) |
|  | Normal | 70099 (47.58) |
|  | Overweight | 36562 (24.82) |
|  | Obese | 14705 (9.98) |
| Birth Order | 1-2 | 104891 (71.19) |
|  | 3-4 | 34623 (23.5) |
|  | Higher | 7818 (5.31) |
| Terminated Pregnancy | No | 124872 (84.76) |
|  | Yes | 22460 (15.24) |
| EIBF | No | 82652 (56.1) |
|  | Yes | 64680 (43.9) |
| Low Birth Weight | No | 123423 (83.77) |
|  | Yes | 23909 (16.23) |
| Stunting | No | 99361 (67.44) |
|  | Yes | 47971 (32.56) |
| Wasting | No | 120281 (81.64) |
|  | Yes | 27051 (18.36) |
| Underweight | No | 105065 (71.31) |
|  | Yes | 42267 (28.69) |
| AOFOCM | No | 72910 (49.49) |
|  | Yes | 74422 (50.51) |
| MCFOCM | No | 111006 (75.34) |
|  | Yes | 36326 (24.66) |

Table S2: Association of confounders with the exposure variable (Pre-matching).

| **Variables** | **Level** | **Low Birth Weight** | |  |
| --- | --- | --- | --- | --- |
|  |  | Yes  23909 (16.23) | No  123423 (83.77) | p-value  (Chi-square) |
| Country | Bangladesh | 209 (13.67) | 1320 (86.33) | <0.001 |
|  | India | 23247 (16.32) | 119208 (83.68) |  |
|  | Maldives | 212 (12.86) | 1437 (87.14) |  |
|  | Nepal | 138 (11.94) | 1018 (88.06) |  |
|  | Pakistan | 103 (18.97) | 440 (81.03) |  |
| Mother’s Age | <20 | 867 (20.79) | 3303 (79.21) | <0.001 |
|  | 20-34 | 20802 (16.26) | 107113 (83.74) |  |
|  | >=35 | 2240 (14.69) | 13007 (85.31) |  |
| Mother’s Education | No education | 4866 (18.65) | 21229 (81.35) | <0.001 |
|  | Primary | 3212 (17.96) | 14669 (82.04) |  |
|  | Secondary | 12755 (16) | 66946 (84) |  |
|  | Higher | 3076 (13) | 20579 (87) |  |
| Wealth Index | Poorest | 6346 (18.73) | 27532 (81.27) | <0.001 |
|  | Poor | 5632 (16.8) | 27892 (83.2) |  |
|  | Middle | 4611 (15.32) | 25495 (84.68) |  |
|  | Rich | 4084 (15.01) | 23131 (84.99) |  |
|  | Richest | 3236 (14.31) | 19373 (85.69) |  |
| Sex of Child | Male | 11853 (15.04) | 66965 (84.96) | <0.001 |
|  | Female | 12056 (17.6) | 56458 (82.4) |  |
| ANC | <4 | 9922 (17.65) | 46292 (82.35) | <0.001 |
|  | >=4 | 13987 (15.35) | 77131 (84.65) |  |
| Take Iron Tablet | No | 2923 (18.71) | 12698 (81.29) | <0.001 |
|  | Yes | 20986 (15.93) | 110725 (84.07) |  |
| C-Section | No | 18313 (16.23) | 94496 (83.77) | 0.921 |
|  | Yes | 5596 (16.21) | 28927 (83.79) |  |
| Place of Residence | Urban | 5143 (15.65) | 27710 (84.35) | 0.001 |
|  | Rural | 18766 (16.39) | 95713 (83.61) |  |
| Exposed to Media | No | 6887 (18.69) | 29955 (81.31) | <0.001 |
|  | Yes | 17022 (15.41) | 93468 (84.59) |  |
| BMI | Underweight | 5343 (20.58) | 20623 (79.42) | <0.001 |
|  | Normal | 11434 (16.31) | 58665 (83.69) |  |
|  | Overweight | 5145 (14.07) | 31417 (85.93) |  |
|  | Obese | 1987 (13.51) | 12718 (86.49) |  |
| Birth Order | 1-2 | 17179 (16.38) | 87712 (83.62) | 0.007 |
|  | 3-4 | 5433 (15.69) | 29190 (84.31) |  |
|  | Higher | 1297 (16.59) | 6521 (83.41) |  |
| Terminated Pregnancy | No | 20046 (16.05) | 104826 (83.95) | <0.001 |
|  | Yes | 3863 (17.2) | 18597 (82.8) |  |
| EIBF | No | 14502 (17.55) | 68150 (82.45) | <0.001 |
|  | Yes | 9407 (14.54) | 55273 (85.46) |  |

Table S3: Covariate balance before and after matching.

| **Variables** | **Pre-Match Treated** | **Pre-Match Control** | **Pre-Match SMD** | **Post-Match Treated** | **Post-Match Control** | **Post-Match SMD** |
| --- | --- | --- | --- | --- | --- | --- |
| distance | 0.1705 | 0.1607 | 0.2628 | 0.1705 | 0.1705 | 0.0001 |
| mother_age | 1.0574 | 1.0786 | -0.0596 | 1.0574 | 1.0543 | 0.0087 |
| mothers_edu0 | 0.2035 | 0.1720 | 0.0783 | 0.2035 | 0.2025 | 0.0026 |
| mothers_edu1 | 0.1343 | 0.1189 | 0.0454 | 0.1343 | 0.1305 | 0.0113 |
| mothers_edu2 | 0.5335 | 0.5424 | -0.0179 | 0.5335 | 0.5385 | -0.0100 |
| mothers_edu3 | 0.1287 | 0.1667 | -0.1137 | 0.1287 | 0.1286 | 0.0002 |
| wealth_index1 | 0.2654 | 0.2231 | 0.0959 | 0.2654 | 0.2681 | -0.0062 |
| wealth_index2 | 0.2356 | 0.2260 | 0.0226 | 0.2356 | 0.2359 | -0.0009 |
| wealth_index3 | 0.1929 | 0.2066 | -0.0347 | 0.1929 | 0.1916 | 0.0031 |
| wealth_index4 | 0.1708 | 0.1874 | -0.0441 | 0.1708 | 0.1694 | 0.0038 |
| wealth_index5 | 0.1353 | 0.1570 | -0.0632 | 0.1353 | 0.1349 | 0.0013 |
| sex_of_child | 1.5042 | 1.4574 | 0.0936 | 1.5042 | 1.5039 | 0.0007 |
| anc | 0.5850 | 0.6249 | -0.0810 | 0.5850 | 0.5861 | -0.0022 |
| iron_tab | 0.8777 | 0.8971 | -0.0591 | 0.8777 | 0.8804 | -0.0080 |
| c_section | 0.2341 | 0.2344 | -0.0008 | 0.2341 | 0.2321 | 0.0046 |
| place_resid | 1.7849 | 1.7755 | 0.0229 | 1.7849 | 1.7895 | -0.0111 |
| media_expose | 0.7119 | 0.7573 | -0.1001 | 0.7119 | 0.7125 | -0.0012 |
| bmi | 2.1579 | 2.2935 | -0.1570 | 2.1579 | 2.1520 | 0.0069 |
| terminated_preg | 0.1616 | 0.1507 | 0.0296 | 0.1616 | 0.1581 | 0.0093 |
| eibf | 0.3935 | 0.4478 | -0.1113 | 0.3935 | 0.3919 | 0.0032 |
| countryBangladesh | 0.0087 | 0.0107 | -0.0210 | 0.0087 | 0.0087 | 0.0000 |
| countryIndia | 0.9723 | 0.9658 | 0.0394 | 0.9723 | 0.9723 | 0.0000 |
| countryMaldives | 0.0089 | 0.0116 | -0.0296 | 0.0089 | 0.0089 | 0.0000 |
| countryNepal | 0.0058 | 0.0082 | -0.0327 | 0.0058 | 0.0058 | 0.0000 |
| countryPakistan | 0.0043 | 0.0036 | 0.0113 | 0.0043 | 0.0043 | 0.0000 |

SMD: Standardized Mean Difference

Table S4: Pseudo R-square values with likelihood ratio (LR) test of goodness of fit obtained from logit models for pre-match and post-match data.

|  | **Pseudo R-square** | **LR test statistic** | **p-value** |
| --- | --- | --- | --- |
| Pre match | 0.0112 | 1470.2 | <0.00001 |
| Post match | 0.0001 | 7.3027 | 0.9975 |

Matched sample: 47,818

Table S5: Effect modification by country.

| **Variables** | **Country** | **AOR** | **95% CI** | **P-value** |
| --- | --- | --- | --- | --- |
| Stunting | Bangladesh | 2.615 | (1.583, 4.319) | 0.0002 |
|  | India | 1.457 | (1.402, 1.514) | <0.0001 |
|  | Maldives | 1.517 | (0.947, 2.43) | 0.0832 |
|  | Nepal | 3.168 | (1.828, 5.49) | <0.0001 |
|  | Pakistan | 3.254 | (1.695, 6.245) | 0.0004 |
| Wasting | Bangladesh | 1.593 | (0.769, 3.3) | 0.2106 |
|  | India | 1.297 | (1.239, 1.357) | <0.0001 |
|  | Maldives | 1.346 | (0.752, 2.41) | 0.3174 |
|  | Nepal | 2.891 | (1.23, 6.792) | 0.0149 |
|  | Pakistan | 3.96 | (1.254, 12.512) | 0.0190 |
| Underweight | Bangladesh | 3.097 | (1.842, 5.206) | <0.0001 |
|  | India | 1.782 | (1.713, 1.854) | <0.0001 |
|  | Maldives | 2.199 | (1.346, 3.593) | 0.0017 |
|  | Nepal | 4.4 | (2.364, 8.188) | <0.0001 |
|  | Pakistan | 2.483 | (1.205, 5.117) | 0.0137 |
| AOFOCM | Bangladesh | 2.901 | (1.874, 4.492) | <0.0001 |
|  | India | 1.574 | (1.516, 1.634) | <0.0001 |
|  | Maldives | 1.722 | (1.145, 2.59) | 0.0091 |
|  | Nepal | 4.547 | (2.69, 7.688) | <0.0001 |
|  | Pakistan | 2.71 | (1.486, 4.941) | 0.0011 |
| MCFOCM | Bangladesh | 2.486 | (1.414, 4.368) | 0.0016 |
|  | India | 1.763 | (1.692, 1.837) | <0.0001 |
|  | Maldives | 1.73 | (1.019, 2.937) | 0.0423 |
|  | Nepal | 3.898 | (2.031, 7.482) | <0.0001 |
|  | Pakistan | 3.483 | (1.57, 7.73) | 0.0022 |
